# Meaningful Test and Evaluation of Indoor Localization Systems in Semi-Controlled Environments

**DOI:** 10.3390/s22072797

**Published:** 2022-04-06

**Authors:** Jakob Schyga, Johannes Hinckeldeyn, Jochen Kreutzfeldt

**Affiliations:** Institute for Technical Logistics, Hamburg University of Technology, 21079 Hamburg, Germany; johannes.hinckeldeyn@tuhh.de (J.H.); jochen.kreutzfeldt@tuhh.de (J.K.)

**Keywords:** indoor localization, test and evaluation, methodology, benchmarking

## Abstract

Despite their enormous potential, the use of indoor localization systems (ILS) remains seldom. One reason is the lack of market transparency and stakeholders’ trust in the systems’ performance as a consequence of insufficient use of test and evaluation (T&E) methodologies. The heterogeneous nature of ILS, their influences, and their applications pose various challenges for the design of a methodology that provides meaningful results. Methodologies for building-wide testing exist, but their use is mostly limited to associated indoor localization competitions. In this work, the *T&E 4iLoc Framework* is proposed—a methodology for T&E of indoor localization systems in semi-controlled environments based on a system-level and black-box approach. In contrast to building-wide testing, T&E in semi-controlled environments, such as test halls, is characterized by lower costs, higher reproducibility, and better comparability of the results. The limitation of low transferability to real-world applications is addressed by an application-driven design approach. The empirical validation of the *T&E 4iLoc Framework*, based on the examination of a contour-based light detection and ranging (LiDAR) ILS, an ultra wideband ILS, and a camera-based ILS for the application of automated guided vehicles in warehouse operation, demonstrates the benefits of T&E with the *T&E 4iLoc Framework*.

## 1. Introduction

In many areas of daily life, we encounter location-based services (LBS), such as for navigation [1], delivery tracking [2], or contact tracing [3]. A localization system determines the location of an entity and therefore enables LBS. Indoor localization and indoor localization systems (ILS) are referred to when localization is performed indoors. Mautz [4] presents several applications of indoor localization, such as the tracking of patients and equipment in hospitals, locating firefighters in a burning building, or optimizing material flow in warehouses.

Although ILS are attributed an enormous potential, their application in practice is rare. In contrast to outdoor applications, which are mostly based on the Global Positioning System (GPS), no technology has prevailed so far. Potortì et al. [5] point out the lack of standardized concepts, procedures, and metrics in test and evaluation of indoor localization systems as a reason for low market transparency, and consequently low trust of stakeholders, and a slow adoption rate. Likewise, Pasricha [6] share this view and name the definition of application-domain-specific requirements as one of the key open research challenges in the field of indoor localization. Lymberopoulos et al. [7] point out that to promote the adoption of ILS, it is essential for test and evaluation (T&E) to be meaningful.

**Figure 1:**
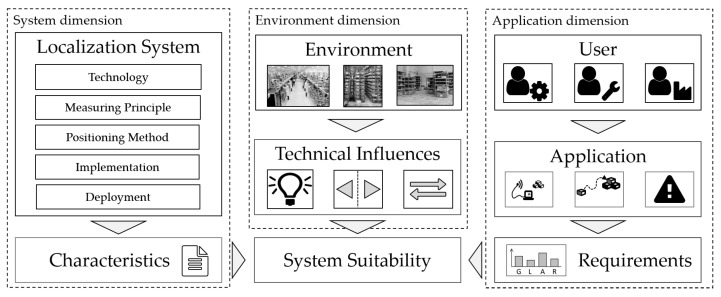
Matching requirements and localization systems—a multi-dimensional problem.

What does it mean for test and evaluation to be meaningful, and what is the reason that localization systems are rarely subjected to meaningful test and evaluation? Meaningfulness in test and evaluation is characterized by the significance of the results to the stakeholders involved. To be meaningful to system developers, the test and evaluation results must be reproducible and comparable to other systems and experiments. From the perspective of an end-user or system integrator, test and evaluation serves to determine a system’s suitability for a particular task. The main reason that ILS are rarely subject to meaningful test and evaluation is that test and evaluation of indoor localization systems is an extremely challenging task [8]. Mautz [4] terms the issue of finding the optimal localization system for an application as a multi-dimensional problem. Figure 1 illustrates this problem, with the dimensions explained as follows.

- System dimension (Figure 1, left): The characteristics of a localization system depend on a variety of factors, such as the underlying technology (e.g., Bluetooth, vision), the measuring principle (e.g., time-of-flight, received signal strength), the positioning method (e.g., fingerprinting, pedestrian dead reckoning, multilateration), the implementation (hard- and software), and the system deployment (e.g., position and amount of anchor nodes), while data fusion techniques are commonly applied on different levels [9]. Deployment is of particular importance for infrastructure-based systems.
- Environment dimension (Figure 1, middle): The system’s performance is affected by technical influences, such as occlusion, reflection, or radio interference which in turn are a consequence of the environment [10]. The environment for the application of ILS can vary widely, ranging from underground mines to multi-story office buildings or warehouses. The impact of the influencing factors on system performance depends on the system characteristics. Since the technology of indoor localization systems is heterogeneous, the dependencies on influencing factors likewise vary.
- Application dimension (Figure 1, right): To find a suitable system, the system performance must meet the requirements of the user. The type and level of requirements vary depending on the application and user. Sixteen potential user requirements, such as accuracy, latency, and integrity, are described by Mautz [4].

To achieve meaningful test and evaluation results, the dimensions described must be taken into account. Significant spatial, financial, and organizational resources, as well as extensive metrology, statistical, data processing, and application domain expertise, are required for meaningful testing and evaluation [11]. Pasricha [6] point out that as a consequence, in practice, mostly simple proof-of-concept tests are performed.

Methodologies are essential to support systematic test and evaluation and thus increase the meaningfulness of results. Methodologies for building-wide test and evaluation, such as the EvAAL Framework [12] and the EVARILOS Benchmarking Handbook [13], have been proposed. The ISO/IEC 18305 International Standard [14] was presented in 2016 to define a standardized procedure for test and evaluation of indoor localization systems with a focus on the end-user. Yet, publicly documented use of the named methodologies for test and evaluation is mostly limited to associated indoor localization competitions [7,15,16,17]. We published a work-in-progress paper on the topic of application-driven test and evaluation of indoor localization systems for warehouses for the Conference on Indoor Positioning and Indoor Navigation (IPIN) in 2021 [18]. This work expands the previously presented findings substantially by generalizing the methodology and by further elaborating all elements of the paper and the framework’s components.

The main reason for the low adoption of methodologies are the high cost of building-wide testing, especially for infrastructure-based ILS. For many localization systems and application environments, building-wide testing is required. Nonetheless, there are others, where the presence of rooms or other fixed structures is of minor importance or can be physically modeled. For such cases, tests are oftentimes performed in test halls that can be modified, for example, by the placement of certain objects [19,20,21,22,23]. In contrast to building-wide testing, this is considered a semi-controlled test environment, whereby semi-controlled means that some influences can be controlled, while others cannot. Since the environment can be controlled to achieve good performance results, the need for a methodology to achieve meaningful results is even more evident. However, a methodology for T&E of ILS in semi-controlled environments does not yet exist.

In this work, the process of application-driven test and evaluation of ILS, the involved stakeholders, and their requirements are first analyzed in Section 2. Furthermore, test and evaluation in practice and existing methodologies are discussed. In Section 3, the *T&E 4iLoc Framework* is presented. The aim of the proposed methodology is to increase meaningfulness for T&E in semi-controlled environments. It consists of several modules for specifying requirements, defining application-dependent scenarios, and for system-level, black-box T&E of indoor localization systems. The empirical validation of the *T&E 4iLoc Framework* is described in Section 4, whereby a camera-based, a light detection and ranging (LiDAR)-based, and a ultra wideband (UWB) ILS are examined, considering the application “Automated Guided Vehicles in Warehouse Operation”. Finally, the proposed methodology is discussed and conclusions are drawn in Section 5.

## 2. Test and Evaluation of Indoor Localization Systems

Test and evaluation is in practice oftentimes carried out by system developers with the purpose of examining the performance of a localization system or its components [6]. Developers are particularly interested in the characteristics of the system itself and the differentiation and quantification of technical influencing factors on the system’s performance. For users, it is paramount how the system performs for a certain task in a given environment. To design a methodology that is meaningful, the stakeholders and their requirements regarding test and evaluation are first examined. Then, it is shown how test and evaluation is performed in practice. Finally, the existing methodologies are discussed in terms of stakeholder requirements for test and evaluation.

### 2.1. Stakeholders and Requirements

**Figure 2:**
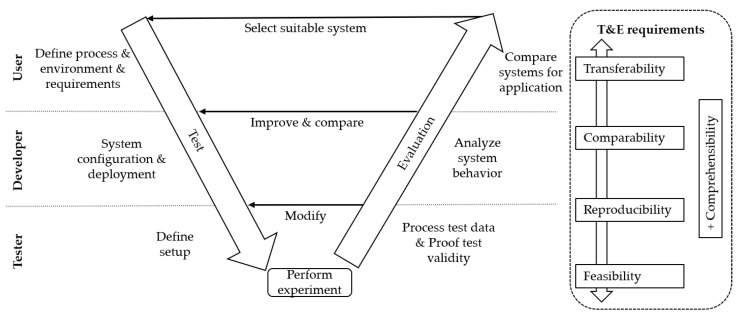
The V-Model—illustration of the application-driven T&E process with the involved stakeholders, their functions, and requirements.

Test and evaluation of localization systems can have a number of objectives, while the requirements depend on the stakeholders’ perspective in the test and evaluation process [24]. The V-Model, commonly used as a management tool for software development and testing, has been adapted to fit the application-driven test and evaluation of localization systems, as shown in Figure 2. The V-Model enables the representation of the process with the associated stakeholders and their functions in different stages over time. Conclusions can be drawn from the evaluation back to the respective test stages. The stakeholders are divided into three categories—users, developers, and testers.

#### 2.1.1. User

System users pursue a localization system to meet the requirements for a certain application. A user can either be an end-user or a system integrator. We consider an example from the logistics domain to clarify this division. An example of a system integrator could be the manufacturer of automated forklifts, while the localization system serves to enable the navigation of the forklift in warehouses. The system integrator builds localization systems into a product. An end-user, on the other hand, could be a warehouse operator who equips the warehouse with a localization system, to enable the tracking of goods and assets. An end-user pursues localization systems either from the system developer or integrated into a higher-level product. Users strive to select the optimal system for their specific needs. They require that test and evaluation results are comparable to results of other systems, environments, or processes. We refer to this requirement as comparability. In addition, it is required that the results are transferable, i.e., that results are reproducible in real-world applications. To support transferability, users have to specify the considered application by defining the processes, environment, and system requirements.

#### 2.1.2. Developer

Developers are soft- or hardware engineers who participate in the advancement of the technology. Sensor and algorithm development often results in prototype localization systems and components that must be tested and evaluated to draw conclusions about the performance. During application-driven test and evaluation, developers must specify the configuration and deployment of the localization system to meet the user’s system requirements, ensuring that the results meet the requirements for comparability and transferability. Test and evaluation results are used by developers to evaluate and improve the system, its configuration, or its deployment. To assess system performance, developers require comparability of the test and evaluation result with other configurations, deployments, and environments. Comparability can only be achieved if test and evaluation results are repeatable and reproducible. Repeatability is the ability to achieve the same results when testing the same system in the same way in the same environment [14], while reproducibility is the ability to achieve the same results when testing the same system in a different environment [13]. In contrast to reproducibility, comparability allows comparison between the performance of systems based on the same or different scenarios. Non-repeatable testing cannot be reproducible. In the following, we will therefore refer to reproducibility, whereby repeatability is included. The reproducibility of tests depends not only on the test and evaluation process, but also on the system itself, and can only be achieved to a certain extent.

#### 2.1.3. Tester

Testers define a concrete test setup, perform experiments, process data, and prove the validity of the results. They have to ensure the abovementioned requirements for transferability, comparability, and reproducibility while at the same time enabling the feasibility of the test and evaluation. Feasibility measures the required spatial, financial, organizational, and human resources required to perform test and evaluation.

In addition, the comprehensibility of the processes and the data at each level of the V-Model must be given for the respective stakeholders. Clearly defined processes, meaningful performance metrics, and documentation increase comprehensibility.

In practice, the stakeholder categories often overlap. In academic research, developers usually test their systems or system components themselves. Companies that are considered users or developers may also perform test and evaluation of the systems. The categories should be considered according to the role that a company, a department, or an individual plays during the process of test and evaluation. The defined requirements, namely transferability, comparability, reproducibility, feasibility, and comprehensibility, are partially conflicting. Fully transferable test and evaluation requires T&E to be performed in the operating warehouse based on the exact processes of the application. This would limit comparability, reproducibility, and feasibility. The defined requirements thus form a field of tension. Test and evaluation must be designed accordingly, while methodologies can be applied to support finding a suitable compromise.

### 2.2. Test and Evaluation in Practice

In the following is presented how test and evaluation of indoor localization systems are performed in practice. The limitations are pointed out, and practical approaches to overcoming these limitations are discussed.

Localization systems and their components are commonly tested and evaluated by developers. The results are often published as experimental validation, e.g., in research papers. While suitable as a proof-of-concept, quantitative results must at least be regarded with caution [6,10]. Adler et al. [25] presented a myriad of testing procedures and evaluation metrics, based on 183 publications from the Indoor Positioning and Indoor Navigation (IPIN) conference of 2010 to 2014. The experiments range from simple office walks to real-world experiments in populated buildings. While absolute positioning accuracy is usually considered the main criterion, there are inconsistencies regarding the respective metric and the determination of the system’s true position. Another survey was published by Bousdar Ahmed et al. [24] in 2020. In contrast to Adler et al. [25], Bousdar Ahmed et al. [24] analyzed the test and evaluation processes according to the characteristics defined in the ISO/IEC 18305 Standard [14]. While oftentimes not specified, the analyzed experiments vary widely regarding the building type, the number of floor levels, the type of motion, the number of evaluation points, the applied accuracy metrics, and the used ground truth. In addition, we analyzed the papers published at the IPIN conference in 2021 to examine the recent usage of methodologies. There were 85 full papers published at the IPIN 2021 conference. Empirical test and evaluation of the absolute localization accuracy was performed in 33 publications. None of the 33 publications is referring to an existing test and evaluation methodology. A table of the papers and their categorization into the underlying technology, ground truth, and test environment is provided in Appendix A. As a consequence of this diversity, comparability and reproducibility are low. Nonetheless, results are summarized in review papers [4,26,27,28] based on hardly comparable quantities [12]. T&E results furthermore lack transferability, as concrete use-cases are rarely considered or specified. For system users, low transferability leads to the consideration or selection of systems and technologies that do not perform in practice as the test and evaluation results indicate [7]. To overcome these limitations, various approaches have been developed, each with its own advantages and disadvantages. The approaches are not mutually exclusive, but complementary.

#### 2.2.1. Simulated Data

Test data can be generated by applying simulation models of the localization system and the environment. Evaluating localization systems and algorithms based on simulation data has the advantage of minimizing the costs of space, time, and equipment for the physical experiment setup while allowing full repeatability of experiments [29]. Nevertheless, simulations are always an abstraction of reality and are therefore object to certain limitations. For example, uncertainties in simulations are usually modeled by Gaussian noise [30]. In reality, measurement uncertainties result from complex interactions of various hardware and software components with the environment and go far beyond. While simulations are a useful tool for developers, they cannot replace experiments in the real world.

#### 2.2.2. Common Datasets

Inspired by the vast growth of robotics and artificial intelligence research, the indoor localization community has introduced benchmarking based on prerecorded datasets [31]. Raw data from localization systems are provided, such as the received signal strength (RSS) for Wi-Fi fingerprinting [32] or image data for visual localization [33]. The generation of raw data is separate from the actual localization algorithm. Hence, test and evaluation at the system level of a localization system cannot be performed. Benchmarking based on shared datasets has proven to be extremely useful to the research community; however, the benefit to the system user, who is mainly interested in how a system works as a whole, is limited.

#### 2.2.3. Indoor Localization Competitions

Since the first IPIN conference in 2010, several competitions of indoor localization systems have been organized. In 2011, the EvAAL competition was held in conjunction with the IPIN conference, with the aim of evaluating indoor localization systems in the same environment with similar conditions [15]. Since then, the competition has been held annually, but was renamed to the IPIN competition in 2014 [34]. The IPIN competition consists of several evaluation tracks focused on different use-cases and technologies. In 2016, for example, a track was designed to evaluate ILS for tracking of robots [35]. In 2017, the PDR Challenge in Warehouse Picking, and in the following year, the xDR Challenge for Warehouse Operation, were organized to evaluate systems for dead reckoning of pedestrians and vehicles in warehouse environments [36]. The PerfLoc competition was held between 2017 and 2018 by the U.S. National Institute of Standards and Technology (NIST), recording data from smartphone sensors [16,17]. The Microsoft Indoor Localization Competition was first held in 2014 in conjunction with the International Conference on Information Processing in Sensor Networks (IPSN) [7]. In 2021, the Microsoft Indoor Localization Competition attracted 1446 participants, competing based on datasets, which were collected by smartphone sensors in 212 different buildings [37]. A comprehensive overview of the characteristics of the aforementioned competitions is given by Ichikari et al. [36]. Competitions lead to good comparability of localization systems for the intended scenario and good transferability for the considered application domain or environment. On the other hand, transferability to other applications and comparability with other test and evaluation results remain limited. In addition, competitions based on the test and evaluation on a system level are costly and hardly scalable [30].

It was shown that quantitative performance results of test and evaluation are often not meaningful due to lack of transferability, comparability, and reproducibility. The approaches presented have been developed to mitigate these limitations, but rarely focus on the system user. Test and evaluation methodologies can be applied to systematize the T&E process and increase the meaningfulness of results.

### 2.3. Test and Evaluation Methodologies

A methodology describes the general strategy for solving a problem, whereby decisions can be made within a set of rules [38]. A methodology incorporates methods and/or procedures and can be incorporated by approaches [38]. A benchmark is a well-defined procedure for conducting an experiment, collecting data, and computing metrics. In the following, the terms methodology and framework are used interchangeably.

The methodologies for test and evaluation of indoor localization systems are presented and discussed regarding the T&E requirements. The systematic comparison is supported by considering the following characteristics.

- System-testing vs. component-testing: In contrast to component-testing, system-testing describes how a localization system performs as a whole. There is no consideration of a localization system’s individual components [14].
- Knowledge about the system’s inner workings: Designing test and evaluation processes considering the inner workings of a system leads to system-specific testing. In black-box testing, the test and evaluation process is designed without considering the inner workings of localization systems [14].
- Application-driven T&E approach: Seltzer et al. [39] present three general approaches for application-driven T&E. For the vector-based approach (1), a system vector, consisting of general performance metrics, is determined by microbenchmarks, while the application vector describes the requirements, which are determined by the application. The dot product of the vectors is considered a meaningful result. In the trace-based approach (2), the actual application influences the benchmark itself. Accordingly, the resulting system vector is application-specific. Finally, the hybrid approach (3) combines the trace-based and the vector-based approach.
- Test environment: In laboratory testing, ideally all relevant environmental influences for the test and evaluation can be controlled. In building-wide testing, a large space of the facility is used [14]. Semi-controlled environments allow partial control of environmental influences.
- Building specifications: In building-wide testing, the type of building has a significant impact on the system performance. Hence, the building specification needs to be considered.
- Provision of scenarios: Scenarios define conditions for an experiment.
- Entity to be localized/tracked (ELT): Requirements or considerations regarding the type of ELT.
- Ground truth specification: Requirements or considerations regarding the ground truth. The ground truth provides a location estimate that is considered to be the true location of an ELT. Commonly, a reference system with significantly higher accuracy, or off-line surveyed evaluation points are applied as ground truth [14].
- Specification of path and test points: The system accuracy is usually determined by comparing the location estimate with the ground truth at test points along a certain path. The number, the position, and the order of test points are relevant for the results [6,7].
- Motion specification: Describes the way in which the ELT moves through the environment.
- Proposed metrics: Various metrics can be used to describe the performance of a localization system.
- Metrics applied for absolute position error: The absolute accuracy is usually considered the main criterion for the quality of an ILS. Various metrics can be considered for describing the accuracy of a localization system.
- Consideration of Influences: Influences on the system performance can be considered for the experiment specification and/or system evaluation.

In the following, we give a brief overview of the existing test and evaluation methods, their documented use, and their main advantages and disadvantages. Table 1 provides a comparative overview of the test and evaluation methodologies by showing some background information, such as the related authors and the characteristics described above.

#### 2.3.1. EvAAL Framework

The EvAAL Framework is a set of rules and metrics for the test and evaluation under complex and realistic conditions [12]. The core criteria of the EvAAL Framework demand a natural movement of a human actor carrying the localization device, a realistic test environment, a realistic measurement resolution, and the third quantile of point Euclidean error as a performance metric. Because the EvAAL Framework is a fairly simple set of rules, its core criteria are often met in practical test and evaluation processes without explicit reference to the methodology. Its strengths consist of a high feasibility and comprehensibility. Only one metric is proposed and influences are not considered further, which limits comparability with other experiments and the transferability to real-world applications that differ from the test case. The EvAAL Framework is well suitable for use in competition. However, the results provide little insight into the system behavior and performance beyond the specific test case.

#### 2.3.2. EVARILOS Benchmarking Handbook

The final version of the EVARILOS Benchmarking Handbook was published in 2015 as a result of the project EVARILOS [10,13]. The EVARILOS Benchmarking Handbook defines a scheme for specifying experiments and evaluating the experiments based on several metrics. The application domain is taken into account when specifying experiments and calculating the final performance score according to the application requirements. In addition to point accuracy, the EVARILOS Benchmarking Handbook also considers system latency, energy efficiency, and robustness to changes in radio interference and environment. The EVARILOS Benchmarking Handbook is designed thoroughly, taking into account various metrics and influences, leading to good comparability and reproducibility. Application-specific definition of scenarios and score calculation is enabled. Although the EVARILOS Benchmarking Handbook is sophisticated and several tools are provided [11,40], the methodology has only been applied in the EVARILOS project itself, the EVARILOS Open Challenge [41], the EvAAL competition, and the Microsoft Indoor Localization Competition in 2014 [42].

#### 2.3.3. ISO/IEC 18305:2016 International Standard

Building on the findings of EVARILOS, the ISO/IEC 18305:2016 International Standard was published in 2016 to define terms, scenarios, metrics, and reporting requirements, and to provide considerations for test and evaluation of generic localization systems [14]. The ISO/IEC 18305 is focused on a system-level, black-box, and building-wide test and evaluation approach. Buildings are categorized in five types, such as underground mines and warehouses, and ELT are categorized in “object”, “person”, and “robot”. Different mobility modes for the ELT-type “person” are defined, such as backward walking and sidestepping. The building types, ELT, and mobility modes are combined to 14 test scenarios, without further consideration of the “robot” class. The standard has been discussed in several publications [5,24,43]. However, the publicly documented use of the ISO/IEC 18305 is limited to the PerfLoc competition [16]. Test and evaluation according to the ISO/IEC 18305 leads to meaningful results for both the system developer and the user.

Many efforts have been made to address the problem of meaningful test and evaluation of indoor localization systems. The approaches range from simple proofs-of-concept to extensive experiments in real-world environments in conjunction with competitions based on open datasets. Methodologies aim to systematize test and evaluation, thereby increasing the benefits for the stakeholders. For all methodologies presented, the high costs and effort for building-wide testing are a major limiting factor.

## 3. Test and Evaluation with the *T&E 4iLoc Framework*

In the following, the *T&E 4iLoc Framework* is presented. Unlike existing methodologies for black-box system level, the *T&E 4iLoc Framework* is not designed for building-wide testing. Especially for RF-based systems, the building type, and structures are of great importance to the system performance. However, building-wide testing has the disadvantages of low reproducibility, comparability, and feasibility. In practice, test and evaluation of localization systems is often performed in test halls [19,20,21,22,23]. In this work, the term semi-controlled test environment is introduced as a category for test environments for T&E of indoor localization systems. Semi-controlled test environments are indoor facilities, such as test halls, large rooms, or large open spaces in a building, which provide an open test area with a modifiable environment. There are usually various constraints on the control of the environment, such as the facility itself, the available objects, or the deployment of system infrastructure. It is assumed that in semi-controlled test environments, T&E can be performed with some degree of similarity in terms of factors influencing the system performance. A major challenge for T&E in such environments is to produce results that are meaningful to both system developers and users. The following guidelines were established to incorporate the results from the previous analysis of test and evaluation (Section 2) into the *T&E 4iLoc Framework*.

1. Application focus: Test and evaluation that is meaningful to a system user can only be performed with respect to an application. The heterogeneity of indoor localization applications must be considered.
2. Technology openness: A system user is not interested in the inner mechanisms of a localization system as long as it works according to the system requirements. Consequently, an application-driven methodology must be open to different localization system technologies.
3. Stakeholder focus: System developers and testers require different information from a localization system than system users. A methodology should consider the needs of all stakeholders involved, while a stakeholder should only receive relevant information.
4. Modular design: To find broad acceptance and thus increase comparability, a methodology must be practicable for system users and developers from academic research and industry. Modularity supports feasibility for different stakeholders and use cases while ensuring comparability through clearly defined processes and interfaces.

First, considerations of the *T&E 4iLoc Framework* are stated with respect to the previously discussed characteristics of test and evaluation. Then, the framework architecture and components are explained. An overview of the terminology can be found in Appendix B.

### 3.1. Characteristics

The characteristics of the *T&E 4iLoc Framework* are discussed to provide a comprehensible overview and enable the comparison to the previously presented methodologies in Table 1.

#### 3.1.1. System-Testing vs. Component-Testing and Knowledge about the System’s Inner Workings

The performance of the components of a localization system or the system’s inner workings are not relevant from a system user’s point of view as long as the system meets the requirements. Hence, a system-level and black-box approach is used. The output of the localization system is in the form of absolute position and/or heading data with timestamp.

#### 3.1.2. Application-Driven T&E Approach

The *T&E 4iLoc Framework* is based on a hybrid approach according to Seltzer et al. [39] with the goal of determining the suitability of a localization system for an application. Consequently, it influences both testing and evaluation.

#### 3.1.3. Test Environment and Building Specifications

As previously mentioned, the *T&E 4iLoc Framework* is designed for T&E in semi-controlled environments. It is required that a rectangular test area is provided in an open space. A test area of at least 50 m${}^{2}$ is suggested to enable the guidance of the ELT, object placement, and evaluation of location-dependent errors. This number is based on practical experience for T&E at the Institute for Technical Logistics. Depending on the localization system to be tested and the application under consideration, this value can also be fallen below.

#### 3.1.4. Provision of Scenarios

The provision of predefined test scenarios increases reproducibility and comparability. However, the transfer to a different practical application is limited. Hence, functions are provided to allow flexible but systematic definition of application-dependent test scenarios. In addition, a reference scenario is provided.

#### 3.1.5. ELT and Motion Specification

ELT and its motion can be specified flexibly depending on the application. The entity classes “person”, “object”, and “robot” are adopted as reference classes from the ISO/IEC 18305 [14]. In addition, “vehicles”, such as manually guided industrial trucks, are considered an entity class.

#### 3.1.6. Ground Truth Specification

The *T&E 4iLoc Framework* requires a reference localization system. For off-line surveyed points, no timestamp is provided automatically for ground truth data, which is of particular importance for dynamic measurements. In addition, a reference system provides continuous localization data, which is relevant for system developers and testers to analyze system behavior and to prove test validity. As specified in the ISO/IEC 18305 [14], the ground truth accuracy should be at least one order of magnitude higher than that of the localization system.

#### 3.1.7. Specification of Path and Test Points

The heading of a localization system can have a significant influence on the localization accuracy. Hence, poses (x,y,yaw) are considered instead of points (x,y). A path is defined by evaluation poses, which are traversed by the ELT one after the other. The suggested number of evaluation poses is one per square meter. This number is not fixed and should be adjusted to reach statistically significant results for the localization system, test environment, and the test and evaluation procedure. Guidelines for the selection of evaluation points are provided by de Poorter et al. [44]. The poses can be selected based on various criteria, such as random grid-based sampling or by defining an application-dependent path.

#### 3.1.8. Proposed Metrics and Metrics Applied for Absolute Position Error

The localization accuracy is considered the most important performance characteristic of a localization system. Unlike the EVARILOS Benchmarking Handbook [13] and the ISO/IEC 18305 [14], the *T&E 4iLoc Framework* focuses only on localization accuracy. Metrics other than localization accuracy are not precluded. However, for these, reference is made to the considerations provided in the EVARILOS Benchmarking Handbook [13] and ISO/IEC 18305 [14]. The *T&E 4iLoc Framework* considers horizontal position (*x* and *y*), vertical position (*z*), and heading (yaw), resulting in four degrees-of-freedom (4-DoF). A comprehensive list of the accuracy metrics considered can be found in Section 3.8.

#### 3.1.9. Consideration of Influences

Influences are taken into account by defining scenarios. Analysis of influences on system performance is enabled by comparing performance metrics from different scenarios, e.g., by calculating sensitivity values, as suggested in the EVARILOS Benchmarking Handbook [11], or by multifactor analysis [45]. In this work, the analysis of influences is not addressed in depth.

### 3.2. Architecture

**Figure 3:**
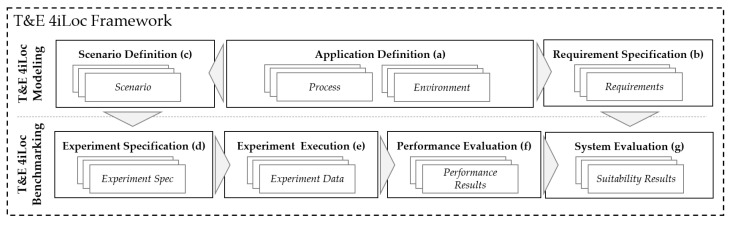
Architecture of the *T&E 4iLoc Framework*.

The *T&E 4iLoc Framework* is designed in a modular way, consisting of seven function modules ((a)–(g)). Each module consists of several functions that result in output data that are further processed by subsequent modules. The framework architecture is shown in Figure 3.

The modules are grouped into two procedures. The *T&E 4iLoc Modeling Procedure* contains the *Application Definition*, *Scenario Definition*, and the *Requirement Specification*. The *T&E 4iLoc Benchmarking Procedure* contains the *Experiment Specification*, *Experiment Execution*, *Performance Evaluation*, and *System Evaluation*. The division into the two procedures enables the distinction between application-dependent T&E by using both, and application-independent T&E, by using only the *T&E 4iLoc Benchmarking Procedure*.

Application-dependent T&E is enabled by the *T&E 4iLoc Modeling Procedure*. First, the application is defined by describing the *Processes* and the *Environment* as part of the *Application Definition*. Application-dependent evaluation is achieved by defining system *Requirements*, as a result of the *Requirement Specification*. On the other hand, testing is influenced by defining a *Scenario* as part of the *Scenario Definition*. The *T&E 4iLoc Modeling Procedure* leads to a testbed-independent abstraction of an application for the test and evaluation.

The *T&E 4iLoc Benchmarking Procedure* can be based on a *Scenario* and *Requirements* for application-dependent T&E, or on freely defined scenarios for application-independent T&E. The testbed-independent scenario is transferred to a testbed-dependent *Experiment Spec*. Then, the experiment is performed based on the functions of the *Experiment Execution*, resulting in a set of *Experiment Data* consisting of continuous, timestamped localization and reference data. The calculation of performance metrics and the data visualization is performed in the *Performance Evaluation*. The *Performance Evaluation* is focused on developers and testers and provides a holistic view of the system behavior. Finally, the *System Evaluation* matches *Requirements* and *Performance Results* by providing and comparing the performance metrics relevant to an application.

In the following, the briefly introduced modules ((a)–(g)). of the *T&E 4iLoc Framework* are explained in more detail by defining their functions (1)–(3) and output data.

### 3.3. Application Definition (a)

**Figure 4:**
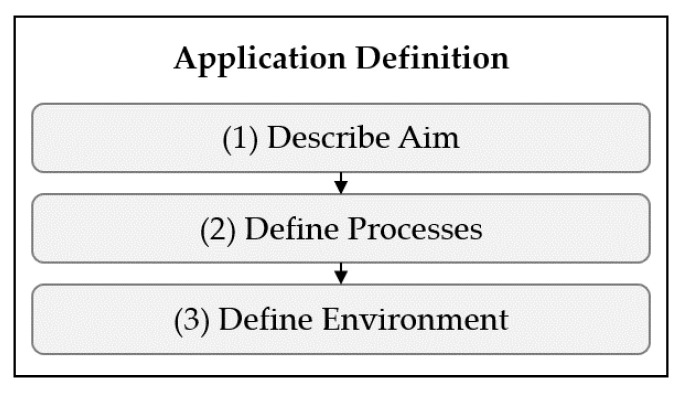
Functions of the module *Application Definition*.

It is critical whether test and evaluation is performed for tracking customers in a supermarket, firefighters in a burning building, or for navigating an autonomous forklift. The goal of the *Application Definition* is to describe exactly what the application or application domain under consideration (AUC) looks like, whereby an application domain contains multiple applications. The functions of the *Application Definition* are shown in Figure 4.

First, the aim of the AUC is described (1). Then, the application is broken down, by defining the respective *Processes* (2) and the *Environment* (3). Depending on the aim of the test and evaluation procedure, it may be reasonable to further divide *Processes* and *Environments*.

To illustrate the functions of the module and their relevance, the example of customer tracking in a supermarket is considered. The aim of the AUC could be to gain transparency of customer behavior by tracking their movements during the shopping process. Exemplary *Processes* are “searching for products”, “picking products”, or “comparing prices”. The *Environment* is defined by describing relevant dynamics and objects in a supermarket. If relevant, the supermarket environment could be further divided, e.g., into shopping and checkout areas.

### 3.4. Requirement Specification (b)

To determine the suitability of a system for an AUC, specific performance requirements must be derived. As discussed in Section 3.1, the *T&E 4iLoc Framework* focuses on performance requirements related to localization accuracy. Hence, the absolute accuracy of the following parameters is considered:

- Horizontal position (x,y);
- Vertical position (*z*);
- Spherical position (x,y,z);
- Heading (yaw).

**Figure 5:**
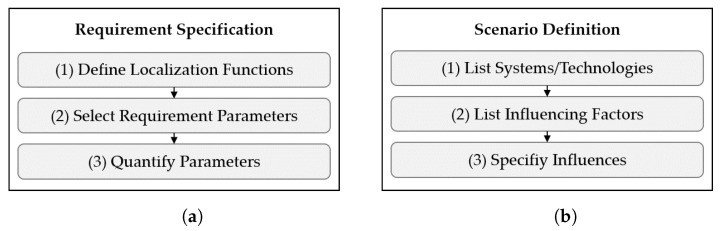
Functions of the module *Requirement Specification* (**a**) and *Scenario Definition* (**b**).

Three functions are defined to specify the requirements (Figure 5a. First, localization functions must be derived (1). Second, relevant requirement parameters are selected (2) by checking whether the accuracy of a parameter is relevant for realizing the localization function. Requirements can be marked as “must” or “shall”. Then, their magnitudes are defined with the respective confidence level (3). The magnitudes depend on the exact localization functions. Thus, generic rules can hardly be defined. In general, the dimensions of the ELT and the size of the region of interest are relevant. The confidence *p* is described by multiple of the standard deviation for a normal distributed measure. To ensure reliable processes, the Six Sigma (6σ) methodology has become established as a quality management tool in the industry [46]. The goal of Six Sigma is to improve the quality of processes to such an extent that only 0.00034% of processes are defective. For the *T&E 4iLoc Framework*, the description by sigma values is adopted to define the following confidence levels for localization data:

- Very low (2σ): p<93.3%;
- Low (3σ): p>93.3%;
- Moderate (4σ): p>99.38%;
- High (5σ): p>99.977%;
- Very high (6σ): p>99.9997%.

It is important to note that the confidence level for each individual position estimate of a system is not equal to the confidence in a localization function, since incorrect location estimates can be compensated at a higher level of the application. While the requirements for tracking people or objects to gain transparency about processes are rather loose, the requirements for controlling robots or vehicles are rather strict.

Considering the example of customer tracking in a supermarket, a localization function could be to determine the horizontal position of customers in the supermarket over time. The relevant requirement parameter is consequently the person’s horizontal position, which should not exceed the shelf width from which the magnitude of the accuracy requirement can be derived.

An application or process can usually be implemented in different ways. It is therefore not possible to prove whether a set of requirements is correct or not until the AUC has been implemented. However, by defining the processes and localization functions, the determination of the performance requirements is comprehensible. *Requirements* can be combined to define the requirements of a process with several localization functions, an application with several processes, or an application domain consisting of multiple applications.

### 3.5. Scenario Definition (c)

A *Scenario* in the *T&E 4iLoc Framework* is an abstraction of an AUC. The aim of a *Scenario* is to enable application-dependent testing with the *T&E 4iLoc Benchmarking Procedure*. It contains a description of the application-dependent influences on the performance of localization systems.

The *Scenario Definition* is based on three functions, shown in Figure 5b. First, systems that are considered to be tested (system under test—SUT) are listed (1). Then, the application-driven influencing factors are listed (2) and specified (3) by matching environmental and process influences with the AUC to define an application-dependent scenario. In contrast to technical influencing factors, such as occlusion or reflection, application-driven influencing factors are derived from the applications’ processes and environment and have a practical character.

A static multi-camera system, as presented by Sun et al. [47], and an UWB localization system are considered for the application of tracking customers in a supermarket. Application-driven influencing factors on the system’s localization accuracy, related to the process, could be the type of entity to be localized, the type of motion of the entity, and the type of path it follows. In terms of the environment, static objects, dynamic objects, and lighting conditions may be considered application-dependent influences on the system’s performance. Finally, to define an application-dependent scenario, the application-driven influencing factors are specified with respect to the AUC. For the given example, the ELT class to be selected is “person”, while the motion type is described by walking at moderate speed. Table 2 gives an example overview of an application-driven scenario for tracking customers in a supermarket.

*Scenarios* enable an application-dependent specification of experiments and can be reused in test and evaluation procedures in different facilities. By specifying experiments based on systematically defined *Scenarios*, reproducibility and comparability are supported, while application-driven influencing factors are considered to increase the transferability of the results.

A reference scenario is provided to establish a standard case for test and evaluation procedures. The reference scenario aims for minimal application-driven influences on generic localization systems. The reference scenario is provided in Table 3. There are neither static nor dynamic objects to be placed. As for the the process influences, the reference scenario requires a robot, which is slowly following a path. The path is defined by randomly distributed evaluation poses. The ELT class “robot” is chosen because the automatic execution leads to an optimal path accuracy and thus increases repeatability of an experiment. The ELT has to be static inside the tolerance area of the evaluation pose. The reference scenario can be extended depending on the influencing factors on the system performance of an SUT.

The *T&E 4iLoc Modeling* is completed by carrying out the *Application Definition*, *Requirement Specification*, and *Scenario Definition*. Application-dependent *Scenarios* and *Requirements* can be used for application-dependent test and evaluation with the *T&E 4iLoc Benchmarking Procedure*. The *T&E 4iLoc Benchmarking Procedure* is elaborated in the following.

### 3.6. Experiment Specification (d)

**Figure 6:**
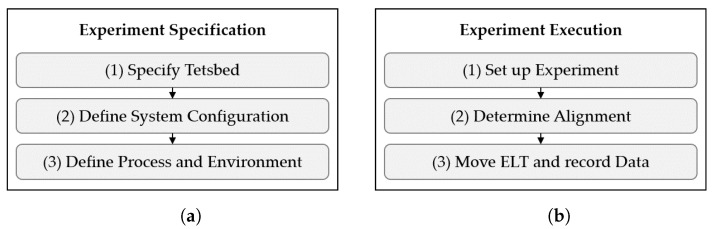
Functions of the module *Experiment Specification* (**a**) and *Experiment Execution* (**b**).

A *Scenario* is transferred into a *Experiment Spec* by the *Experiment Specification*. Unlike a *Scenario*, a *Experiment Spec* is dependent on the testbed. The functions of the *Experiment Specification* are shown in Figure 6a.

First, the testbed is specified (1) by defining the dimensions of the facility, the rectangular test area, available ELT, the environmental control parameters, and the reference system. The provision of photos and layouts of the test facility are suggested. Next, the configuration of the SUT needs to be defined (2) by describing the position of hardware components and the configuration of software parameters. To achieve a realistic setup, it is essential to define the system configuration according to the instructions from the system developer, taking into account the AUC. Finally, the benchmarking process and the environment configuration are defined (3) considering the *Scenario* and testbed specification. The environment is defined by describing the implementation of the environment influencing factors, such as the type and position of static objects. The benchmarking process is defined accordingly, e.g., by specifying the selected ELT, its motion, and path through the calculation of evaluation poses. Tolerance values must be defined as the minimal closeness for reaching an evaluation pose.

**Figure 7:**
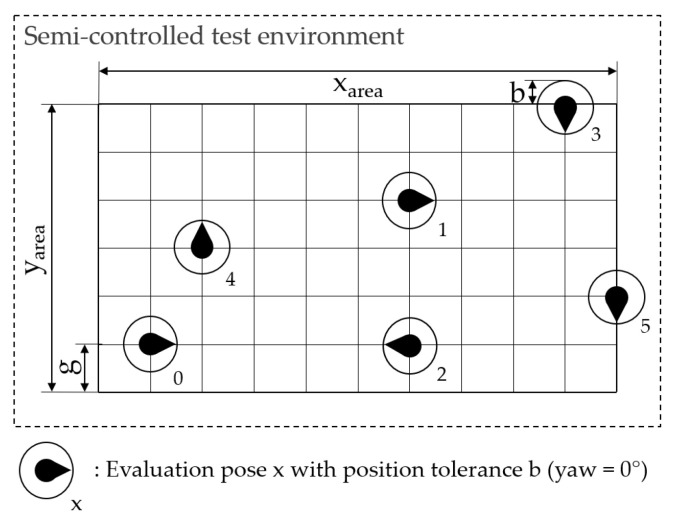
Random grid-based sampled evaluation poses. The arrow points into the heading directions of an evaluation pose.

It is suggested to sample the evaluation poses on a grid with the grid size *g*, which defines the distance between the gridlines. The horizontal position tolerance *b* should be less than half of the grid size to avoid overlapping of tolerance areas. The heading of the evaluation poses should be chosen between a fixed set of options, such as multiples of 90°. This allows for systematic analysis of the location-dependent localization accuracy. For random grid-based sampling, the evaluation poses are randomly selected from the grid points and the options for the heading. Figure 7 illustrates the exemplary result of random grid-based sampling of the evaluation poses on a rectangular test area for six evaluation poses. Depending on the path type defined in the *Scenario*, different sampling methods can be applied. An overview of various sampling methods is provided in the EVARILOS Benchmarking Handbook [13].

The specified testbed, system configuration, environment configuration, and the benchmarking process fully specify an experiment. An experiment can be repeated in the same facility by following the information of an *Experiment Spec*.

### 3.7. Experiment Execution (e)

The *Experiment Execution* serves to create *Experiment Data* based on the *Experiment Spec*. The *Experiment Execution* consists of three functions, as shown in Figure 6b. First, the experiment is set up (1) according to the *Experiment Spec* as follows:

- Setting up the test environment according to the environment specification;
- Deploying and configuring the localization systems according to the system specification;
- Calibrating the reference system;
- Synchronizing the clocks of the reference system and localization system;
- Setting up the ELT to allow its localization with the reference and localization system.

**Figure 8:**
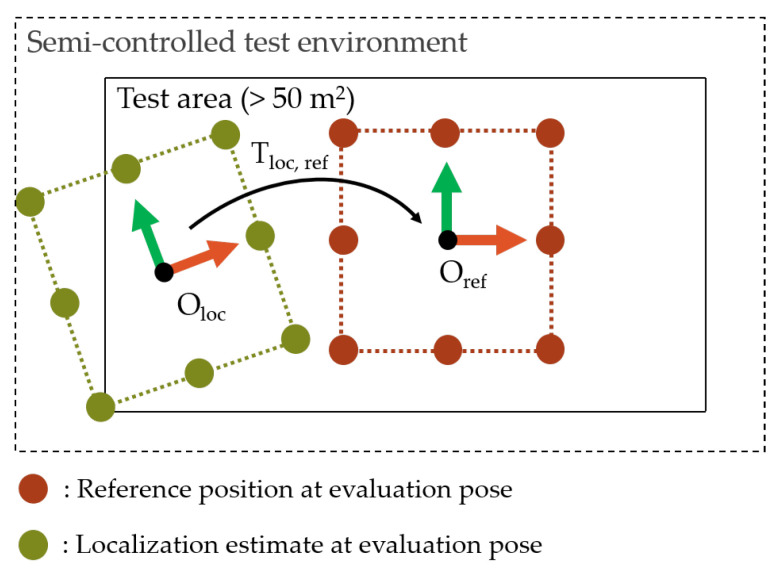
Determination of the transformation matrix $T_{ref,loc}$ between $O_{loc}$ and $O_{ref}$ with the Umeyama alignment [48].

Next, the alignment between the global origins of the coordinate systems of the localization system $O_{loc}$ and the reference system $O_{ref}$ must be determined (2). This is achieved by recording localization and reference data at a number of uniformly distributed points on the test area. It is proposed to compute the transformation matrix $T_{ref,loc}$ by applying the Umeyama alignment [48] without scaling. If the mean pose errors would be eliminated as a bias of the *Experiment Data*, systematic location-dependent errors would be neglected. The alignment is illustrated in Figure 8.

Finally, the *Experiment Data* are recorded from the localization and reference data, while the ELT is passing the evaluation poses in sequence (3). When several localization systems are tested for the same *Scenario*, it is recommended to record the location estimates from multiple systems simultaneously for improving comparability, as suggested by Lymberopoulos et al. [7]. In case of manual guidance, it is recommended to monitor the ELT’s current pose and provide the coordinates of the following evaluation pose. In case of using a robot, it is recommended that the robot obtains the data for navigation from the reference system.

The output data of the *Experiment Execution* consist of the transformation matrix $T_{ref,loc}$ and a list of continuous location estimates and reference data with a synchronized timestamp. The transformation is applied to the localization data in the *Performance Evaluation*.

### 3.8. Performance Evaluation (f)

**Figure 9:**
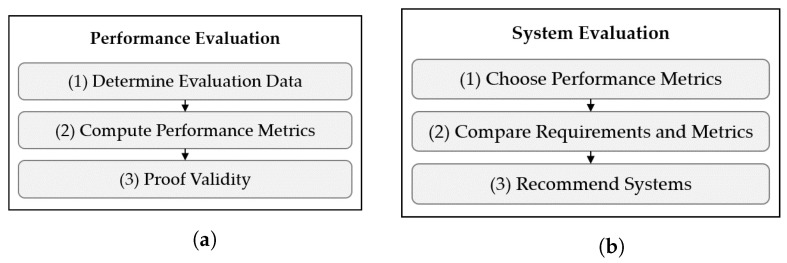
Functions of the module *Performance Evaluation* (**a**) and *System Evaluation* (**b**).

The *Performance Evaluation* aims to produce comparable results based on the *Experiment Data* and to analyze system behavior. The *Performance Results* consist of performance metrics and data for visualization. The functions of the *Performance Evaluation* are presented in Figure 9a.

**Figure 10:**
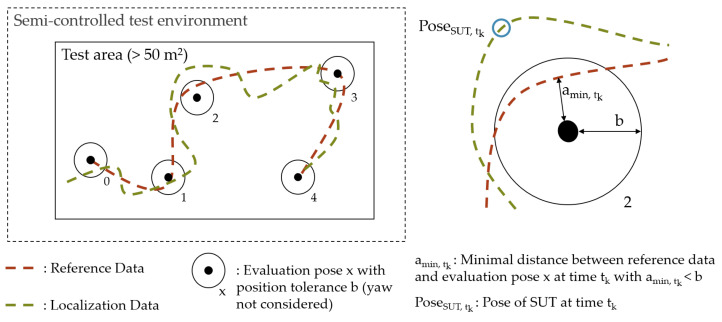
The left side shows a top view of the test area in a semi-controlled test environment, with evaluation poses, interpolated reference data, and aligned localization data. On the right side, a focused view of a test point is shown to illustrate the determination of the *Evaluation Data*.

First, the *Evaluation Data* are determined (1). While the *Experiment Data* consist of the continuous data as measured in the experiment, the *Evaluation Data* are based on the aligned location estimate at the evaluation poses. The alignment of the continuous localization data is achieved by applying the transformation $T_{ref,loc}$. Then, the reference data are linearly interpolated to match the timestamp of the localization data. Finally, the data points corresponding to the position of the reference system closest to the defined evaluation pose are selected. Figure 10 shows the aligned and interpolated localization and reference data for an exemplary experiment. The left side shows a top view of the test area within the semi-controlled environment. Also shown are the evaluation poses (0–4) with their tolerance *b* and the reference and localization data, each defining a trajectory on the horizontal plane. A focused view of test point 2 is shown on the right. A valid data point requires that the reference trajectory passes through the tolerance area. Other tolerances related to the heading or velocity of the ELT may be considered. The data point is selected for the minimum difference $a_{min,t_{k}}$ between evaluation pose and reference data at the time $t_{k}$. The associated data point from the SUT is selected by the corresponding timestamp, resulting in the *Evaluation Data*.

Performance metrics are then calculated (2). The *Performance Evaluation* aims to provide a holistic overview of the system behavior. Therefore, several metrics are calculated based on the error vectors. The 3D error vector $\epsilon_{\left(x,y,z\right)}$ and the absolute 3D error vector $\epsilon_{\left(|x|,|y|,|z|\right)}$ are determined by

(1) $$\epsilon_{(x,y,z),i}=\left(\hat{x_{i}}-x_{i},\hat{y_{i}}-y_{i},\hat{z_{i}}-z_{i}\right),$$

and

(2) $$\epsilon_{(|x|,|y|,|z|),i}=\left(\right|\hat{x_{i}}-x_{i}\left|,\right|\hat{y_{i}}-y_{i}\left|,\right|\hat{z_{i}}-z_{i}\left|\right),$$

whereby $\left(\hat{x_{i}},\hat{y_{i}},\hat{z_{i}}\right)$ denote a location estimate from the SUT and $\left(x_{i},y_{i},z_{i}\right)$ denote the corresponding data point from the reference system, with i=1,2,3,…,n, and *n* equals the amount of evaluation poses. The horizontal, vertical, and spherical position error vectors are determined by computing the Euclidean error of the respective components.

The heading error $\epsilon_{yaw}$ and the absolute heading error $\epsilon_{\left|yaw\right|}$ of the heading estimate $\hat{\psi_{i}}$ and the reference heading $\psi_{i}$ are determined by

(3) $$\epsilon_{yaw,i}=mod\left(\left(\hat{\psi_{i}}+\pi \right),2\pi \right)-\pi ,$$

and

(4) $$\epsilon_{|yaw|,i}=|mod\left(\hat{\psi_{i}}+\pi \right),2\pi )-\pi |,$$

whereby mod(a,b) elaborates the remainder of *a* and *b*.

The following performance metrics are calculated from the vectors of the position error, the absolute position error, the horizontal, vertical, and spherical position error, and the heading error:

- Mean;
- Standard deviation;
- Median;
- RMSE;
- Variances of magnitudes;
- 95th percentile and percentiles related to σ-levels, as presented in Section 3.4.

In practice, many things can go wrong during test and evaluation. Problems with data recording, clock synchronization, calibration, alignment determination, or data processing are common. To ensure the validity of the T&E results, the *Experiment Data* and the *Performance Results* must be carefully checked (3). Visualizations are recommended to gain an understanding of the system behavior. The repeatability or reproducibility of an experiment can be determined by comparing the *Performance Results* from various experiments. In addition, the impact of application-driven influences on the system performance can be examined.

### 3.9. System Evaluation (g)

Potortì et al. [5] criticize metrics defined in the ISO/IEC 18305 for being difficult for the system user to interpret and call for the exclusive use of the 95th percentile. Since applications of localization vary widely, it is not satisfactory for the system user to focus on only one metric. For instance, for customer tracking in a supermarket, it may be sufficient to consider the 3σ-percentile with low position reliability requirements, while for a safety-critical application, such as an autonomous forklift, the percentile corresponding to the 6σ-level might be considered. To resolve this conflict, the *T&E 4iLoc Framework* provides a *System Evaluation* that focuses on the system user and is separate from the *Performance Evaluation* to support comprehensibility for system users without losing relevant information for developers and testers.

The *System Evaluation* is based on the *Requirements* defined in the *Requirement Specification* and on the *Performance Results* with the aim of determining the suitability of a localization system for an AUC. The *Requirements* consist of the description of absolute accuracy and confidence. The module’s functions are shown in Figure 9b. To determine the suitability of a system, the performance metrics corresponding to the requirements are first selected (1). The specified confidence level corresponds to the type of percentile to be considered. Then, the requirements and the performance metrics are compared (2). Finally, a system is considered suitable, if all “must” requirements of an application are met (3). If “shall” requirements are not met, the system is still recommended, but limitations are pointed out.

**Figure 11:**
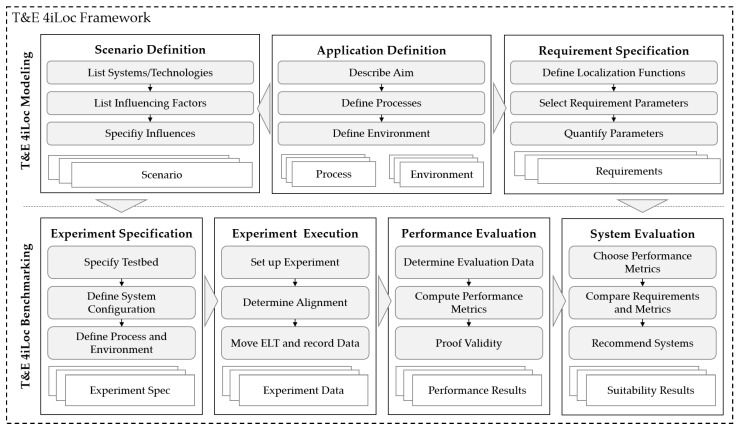
Overview of the *T&E 4iLoc Framework*, with its modules, functions, and output data.

Finally, Figure 11 provides an overview of the *T&E 4iLoc Framework*, including the presented functions for each module. The framework enables the profound determination of a system’s suitability for an application, based on application-driven test and evaluation in a semi-controlled environment. It is open to generic localization systems and focuses on the stakeholder requirements for T&E by providing different function modules. The flexible definition of application-dependent scenarios addresses transferability, while the provision of a reference scenario aims at repeatability and reproducibility.

## 4. Empirical Validation

Meaningful test and evaluation implies that localization systems of various technologies can be analyzed and compared for their performance and suitability for an application. In the following, the *T&E 4iLoc Framework* is used for test and evaluation to examine the framework’s validity, by considering a logistics application.

In logistics processes, everything revolves around the movement of goods and assets. They can only be moved effectively and efficiently if their position is known. Consequently, ILS are an essential tool for improving the understanding and control of processes related to material flow. On the one hand, a logistics application is considered for the empirical validation of the *T&E 4iLoc Framework*, as indoor localization systems are attributed an enormous potential for optimizing material flows. For example, Lee et al. [49] present an application for the tracking of assets, Reinke and Beinschob [50] for enabling automated guided vehicles (AGV) and Macoir et al. [51] for automated inventory management in warehouses. On the other hand, warehouse environments are suitable to be modeled in semi-controlled test environments. In warehouses, the aim is to achieve a high ratio of usable space to floor area. The need for walls, e.g., for fire protection measures, leads to the division of a warehouse into large compartments. Special features in these compartments result from the provision of logistics systems. Test halls resemble warehouse compartments in their basic structure, and the features of a warehouse relevant for localization systems can be modeled on a smaller scale.

In the following, a UWB-based, a camera-based, and a LiDAR-based ILS are exemplary examined for the application of AGVs in warehouse operation, as they represent commonly considered technologies for determining the absolute position of AGVs [52]. The AUC is analyzed and modeled using the *T&E 4iLoc Modeling Procedure*. The performance and suitability of the systems are then determined based on empirical data from the *T&E 4iLoc Benchmarking Procedure*. The *Experiment Data* and the software script used for the performance evaluation are publicly available [53]. The *Jupyter Notebook* file can be used for data comprehension. The test and evaluation procedure is described by explaining the execution of the framework’s function modules.

### 4.1. Application Definition

The goal of the AUC “automated guided vehicle in warehouse operation” is to increase efficiency and safety by flexibly automatizing the material flow in warehouses. Navigation is one of the most essential components of a robot. It serves to find an efficient and collision-free path. The task of navigation is commonly divided into global and local navigation. Global navigation aims at finding the approximate path to reach a certain goal, while local navigation focuses on robotics control [54]. For local navigation, the robot must be aware of its surroundings, which is usually achieved by employing distance sensors. For efficient global navigation, the robot must know its absolute position and heading in the operating environment. In the following, “global navigation” is considered as a process for application-dependent test and evaluation. Nonetheless, many more processes can be defined to enable the efficient use of an AGV in warehouse operations, such as automatic picking and placement of goods or collision avoidance. As for the environment, a general warehouse is considered, characterized by shelving and conveyor systems, floor storage areas, and areas for parking industrial trucks.

### 4.2. Requirement Specification

**Figure 12:**
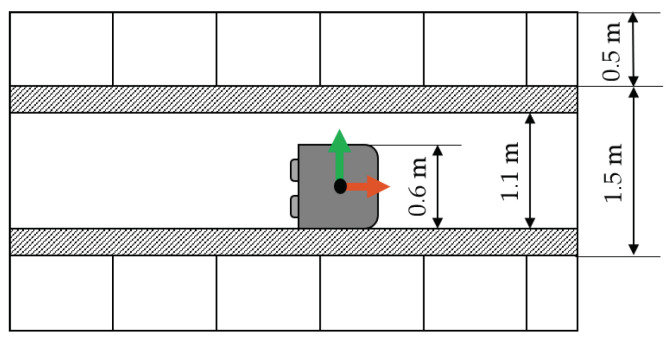
Dimensions of an AGV in an aisle for the quantification of performance requirements.

Identifying the aisle in which the robot is located is considered the critical localization function for the process of “global navigation”. As an AGV is a floor-bound vehicle, the absolute position must be known in the horizontal plane. To quantify this parameter, the dimensions of the robot in an aisle are considered, as shown in Figure 12. The aisle width is 1.50 m, the robot’s width is 0.6 m, and the shelf depth is 0.5 m. To avoid collisions, there is an additional tolerance distance between the AGV and shelves of 0.2 m, resulting in a guidance width of 1.1 m. Even if the robot travels close to the sides of the aisle, the position of the center of the robot must be determined so that it is within the correct aisle. Requirements for the horizontal position accuracy are calculated as the sum of the shelf depth, the tolerance width, and half of the robot’s width, resulting in a horizontal position accuracy requirement of 0.75 m.

In addition, the robot’s heading information can be used to improve global navigation. Hence, the heading is considered a “shall” requirement. For the process of global navigation, the robot’s heading is mainly relevant to identify the side of the aisle it is facing. The robot is assumed to be directed in one of the two directions of the aisle with a tolerance of ±30°. Consequently, the heading requirement for identifying the correct side is ±60°. Unlike for local path planning, safety is less of a concern for global path planning. Therefore, a moderate confidence level of 4σ, i.e., 99.38% is chosen. The requirements for the process “global navigation” are summarized in Table 4.

### 4.3. Scenario Definition

To enable the specification of a meaningful experiment, it is necessary to derive an application-dependent scenario. First, the considered localization systems, i.e., the UWB ILS, LiDAR-based ILS, and camera-based ILS, and their application-dependent influencing factors need to be considered. The location of the multi-layer LiDAR system and the 3D camera system is determined by map matching, i.e., by comparing the sensor data with a previously recorded map of the environment [4,55]. Consequently, the systems’ performance is influenced by the objects in the environment. RF-based localization systems, such as UWB systems, are generally prone to errors in so-called non-line-of-sight (NLOS) conditions. The positioning of the localization system under consideration is based on time-difference-of-arrival measurements [4]. NLOS occurs when the direct line between the transmitting and receiving nodes of a signal is blocked. The material and structure of the occluding objects are important because they influence the signal transmission and reflection [56]. Hence, objects in the environment are considered as a significant influencing factor for defining an application-dependent scenario. To resemble a warehouse environment, shelves with goods of different materials, vehicles, and other logistics equipment are considered. Common warehouse dynamics are mirrored by altering the layout after map recording. Another environmental influence on the performance of the camera-based ILS is the lighting. A mixture of daylight and artificial light is chosen as another application-dependent influence to reflect a typical warehouse environment.

For process influences, the ELT type, the motion, and the path are considered. To reflect the motion of an AGV, the ELT type “robot” is selected. Especially in mixed traffic environments, such as a typical warehouse, robots move at slow velocity. Considering the path, an AGV moves freely on the horizontal plane of a warehouse. For the test scenario, it is required for the robot to move through an aisle. Table 5 summarizes the application-dependent scenario for the AUC.

### 4.4. Experiment Specification

**Figure 13:**
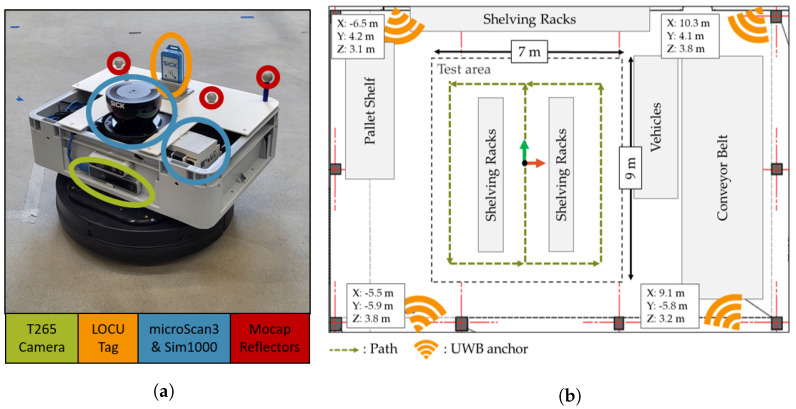
(**a**) Turtlebot2 carrying the localization sensors and motion capture reflectors. (**b**) Schematic overview of the *Experiment Spec*.

The testbed at the Institute for Technical Logistics at Hamburg University of Technology is considered a semi-controlled test environment. By providing a rectangular test area of 63 m${}^{2}$, the framework requirements regarding the size of the test area are met. The environment can be equipped with various objects, such as shelves, pallets, industrial trucks, and other logistics equipment. The testbed features an optical passive motion capture system that enables the determination of 6-DoF reference pose data, with an absolute position accuracy of around 1 mm. Sensors and reflectors are mounted on a TurtleBot2 robotic platform. (Figure 13a). The robot is automatically controlled based on the localization data from the reference system.

The UWB ILS (LOCU [57], SICK AG) consists of four anchors, i.e., fixed nodes, and a localization tag. The anchors are uniformly distributed, avoiding symmetries. The “2D-Mode” is enabled to improve the system’s horizontal position accuracy by limiting the DoF. The LiDAR system consists of a multi-layer safety LiDAR scanner (microScan3 [58], SICK AG) for emission and detection of laser pulses, a control unit (SIM1000 [59], SICK AG), and localization software (LiDAR-LOC [60], SICK AG). The pose of the sensor is determined by comparing scan points with a prerecorded map applying a particle filter [61]. The field of view of the laser scanner is 270° and the localization is supported by an internal inertial measurement unit (IMU). Finally, the localization of the Intel RealSense T265 Tracking Camera [62] is achieved based on a feature-based visual simultaneous localization and mapping (vSLAM) algorithm [63]. The stereo camera is likewise equipped with an IMU and allows identification of loop closures. All systems are deployed according to the installation instructions.

Figure 13b gives a schematic overview of the *Experiment Spec*. The test area is surrounded by typical warehouse objects, such as a pallet shelf, industrial trucks, and a conveyor belt. Two rows of shelving racks are placed inside the test area to build an aisle. A total of 73 evaluation poses are sampled on a grid with 1 m grid length, to guide the robot multiple times through the aisle and around the shelves. The resulting path is depicted in Figure 13b.

### 4.5. Experiment Execution

**Figure 14:**
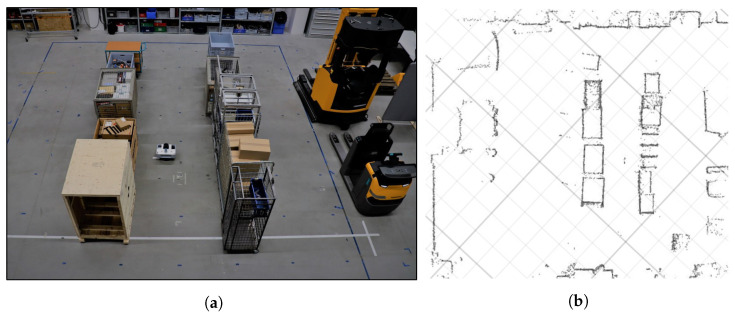
(**a**) Setup of the environment at the Institute for Technical Logistics. (**b**) Recorded map from the LiDAR ILS. The grid with a grid length of 1 m is aligned with the map coordinate system.

The environment and the SUT are set up according to the *Experiment Spec*. A photo of the environment at the Institute for Technical Logistics is shown in Figure 14a. The shelves on the test area are imitated by objects of different materials, resulting in NLOS conditions for the UWB ILS. The reference system is calibrated and synchronized to the localization systems via precision time protocol (PTP) [64] with an offset of less than 0.5 ms. The reference system is set up to accurately locate the origins of the devices on the robot. The Umeyama alignments for the SUTs are then determined based on the measurements from the localization and reference data at nine uniformly distributed positions on the test area. Maps of the environment are recorded for the LiDAR and camera-based ILS. The recorded contour map from the LiDAR ILS is shown in Figure 14b. Then, the sequence of four of the static objects is altered to replicate realistic layout changes in a real warehouse environment.

Finally, the experiment is executed. The localization data are recorded simultaneously from the three SUT and the reference system. The Robot operating System (ROS) [65] is used to control the robot and record the data.

### 4.6. Performance Evaluation

The *Evaluation Data* are computed according to the methods presented in Section 3.8. The *Performance Results* are presented below. The goal is not to provide an exhaustive analysis, but to demonstrate approaches and the need for studying the behavior of the systems. Due to their relevance to the AUC, the focus of the evaluation is on the horizontal components. The performance metrics related to the horizontal error components (*x*, *y*, yaw) are presented in Table 6.

In the following, selected metrics are discussed for the LiDAR-based system. The low mean value and RMSE of the error vector $\epsilon_{x}$ and $\epsilon_{y}$, compared to the respective absolute error vectors, indicate good coordinate alignment and rather low location-dependent inaccuracies. The absolute value of the mean heading error $\epsilon_{yaw}$ is rather high compared to the corresponding standard deviation. A possible reason for this systematic error may be a misalignment of the coordinate axes between the LiDAR sensor and the motion capture reflectors. To compensate for this, the mean of $\epsilon_{yaw}$ could simply be removed as a bias. However, this would also lead to the neglection of location-dependent errors. For a system user, the high percentiles (e.g., the 95th percentile or high sigma levels) of the horizontal position error $\epsilon_{horizontal}$ and the absolute heading error $\epsilon_{\left|yaw\right|}$ are of particular importance.

**Figure 15:**
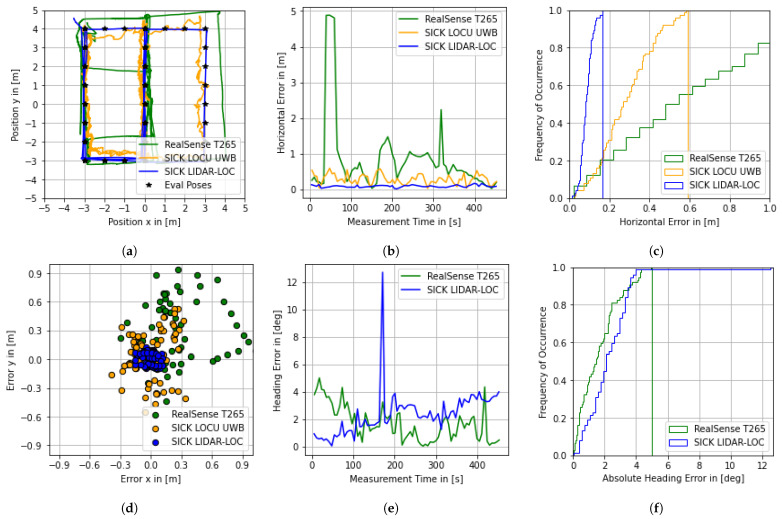
(**a**) Trajectories based on continuous position estimates. (**b**) Horizontal error over measurement time. (**c**) Cumulative distribution histogram of the horizontal error. (**d**) Error scatter. (**e**) Heading error over measurement time. (**f**) Cumulative distribution histogram of the absolute heading error.

In addition, several graphs are shown in Figure 15. The trajectories from Figure 15a are based on the aligned position data of the localization systems, while the other graphs are based on the discrete evaluation data. The trajectory of the camera-based ILS shows significant deviations from the evaluation poses. This is reflected as a leap in the horizontal error graph (Figure 15b). The heading error is not particularly affected by the leaps of the position error. A possible reason for the behavior could be the relocalization of the system in the previously recorded map due to altering the environment after map recording. The position data of the UWB ILS is characterized by a deviation to the center of the test area (Figure 15a) and comparatively high dispersion (Figure 15d). However, there are no major outliers. With the exception of one outlier in the heading estimate, the LiDAR ILS shows reliably good system behavior in terms of horizontal and heading error.

### 4.7. System Evaluation

Finally, the derived performance requirements are matched with the calculated metrics to determine the suitability of a localization system for the process “global navigation”. As previously discussed, the relevant performance requirements are the 4σ-percentiles of the horizontal position error $\epsilon_{horizontal}$ (“must”) and the absolute heading error $\epsilon_{horizontal}$ (“shall”). Table 7 provides a comparison between the requirements and metrics, where the red font color indicates that a requirement is not met. The “must” requirement is met by both the UWB and the LiDAR-based ILS. Hence, both systems are considered suitable for “global navigation”. Since the UWB ILS does not provide heading information, the horizontal position and absolute heading error requirements are only met by the LiDAR system. However, both systems are generally considered suitable for the AUC to enable “global navigation”. For the camera-based ILS, the requirement regarding the horizontal position error is not met.

The *T&E 4iLoc Framework* was designed with the aim to increase meaningfulness of T&E in semi-controlled environments. Requirements were specified and test scenarios designed for the application “AGVs in warehouse operation”. Application-dependent test and evaluation were performed, resulting in several performance metrics for comparison between systems and the determination of the systems’ suitability. Based on the empirical validation, two aspects are highlighted to demonstrate the relevance of the presented methodology. First, the relevance of application-driven test and evaluation is shown by comparison to results from the literature. In an experiment presented by Hausamann et al. [66], analysis of the absolute position accuracy of the RealSense T265 Tracking Camera based on continuous position data yielded a mean horizontal error of around 0.4 m. This is in contrast to a mean error of 0.8 m for application-dependent testing in this work. The data presented by Hausamann et al. [66] were gathered by tracking a person on a significantly smaller test area, without the consideration of aisles or alterations of the environment after map recording. Hence, it may be misleading to consider the test and evaluation results for the application “AGVs in warehouse operation”. The second aspect consists of the need to thoroughly examine the experiment data before drawing final conclusions. Performance metrics are absolutely necessary to compare systems and determine their suitability for an application; however, important characteristics of a system remain hidden if only one metric is looked at. By providing multiple metrics and graphical data representations, possible reasons for localization errors can be identified. Some of these may be relevant for the application, while others should be disregarded.

## 5. Discussion and Conclusions

High heterogeneity of indoor localization technologies, applications, and influences pose multiple challenges for test and evaluation of ILS. By comparing test and evaluation of ILS in practice, the need for methodological approaches becomes evident. Methodologies exist, but they do not fully satisfy stakeholders’ requirements for feasibility, reproducibility, comparability, transferability, and comprehensibility. In this work, the *T&E 4iLoc Framework* was introduced—a methodology for system-level, black-box test and evaluation in semi-controlled environments. Similar to the existing methodologies, the *T&E 4iLoc Framework* is based on a system-level and black-box approach. The main benefits and disadvantages arise from the focus on semi-controlled test environments. By using the *T&E 4iLoc Framework*, the drawbacks of low feasibility, comparability, and reproducibility in building-wide testing can be overcome. On the other hand, building-wide tests have the advantage that the results are highly transferable to real-world applications. To produce results not only meaningful for system developers but also system users, the *T&E 4iLoc Framework* is designed to be application-driven. Hence, consideration of an application or application domain affects both the test and the evaluation of localization systems. However, due to the spatial scaling or the abstraction of environment and application, there are limiting effects with regard to transferability, which require further investigation. Comprehensibility is addressed by providing a modular and process-oriented procedure. The use of the methodology for empirical validation has demonstrated its feasibility. Comparability between systems has been demonstrated for benchmarking the same scenario in the same testbed. Additional experiments are planned to further examine the reproducibility, comparability, and transferability of the test and evaluation results.

Different limitations exist. (1) To achieve meaningful results for T&E in semi-controlled test environments, the AUC and the SUT must be suitable. For example, meaningful test and evaluation of workers in an underground mine based on ultrasonic ILS, as presented by [67], can hardly be achieved. (2) The spatial extent can significantly affect the absolute accuracy of an ILS. Further examination is required, to derive conclusion and potentially metrics for spatial scaling effects. (3) So far, only accuracy metrics that are based on the absolute localization error at discrete evaluation poses are considered. Although other metrics are currently not integrated in the *T&E 4iLoc Framework*, the determination of additional accuracy metrics, such as the repeatability when approaching the same pose several times, is possible. Other metrics, such as Fréchet distance [68] or relative position error [23], can be determined based on the continuous evaluation data. In addition, metrics such as the position error from the direction of the ELT or the velocity-dependent heading error can be determined to analyze system behavior and to test validity. (4) For a holistic evaluation, the consideration of many more factors, such as the sensor weight, maintenance costs, or the system’s integrity, are essential. An overview on possible user requirements is given by Mautz [4].

A web-based application to support systematic test and evaluation using the *T&E 4iLoc Framework* is currently under development and will be released in the near future. Besides the usage of the *T&E 4iLoc Framework* in empirical test and evaluation, it is conceivable to adapt the methodology for approaches such as “simulated data”, “common datasets”, or “indoor localization competitions”. Adoption or adaption of the *T&E 4iLoc Framework* in industry and research are encouraged to increase meaningfulness of test and evaluation results. This is essential to support the application-driven research and development of ILS and increase market transparency, thus, advancing the practical application of ILS.

## Figures and Tables

**Table 1:** Comparisons of methodologies for test and evaluation of indoor localization systems.

	EvAAL Framework [12]	EVARILOS Benchmarking Handbook [13]	ISO/IEC 18305 International Standard [14]
Authors	IPIN International Standards Committee (ISC)	Van Haute et al. [13]	US National Institute of Standards and Technology (NIST)
History remarks	Initially designed for EvAAL competitions. First published in 2017. Applied annually in EvAAL/IPIN competition	Result from EVARILOS project. Final version published in 2015. Applied at EVARILOS Open Challenge, EvAAL competition and Microsoft Indoor Localization Competition in 2014	Based on findings from EVARILOS. First version published in 2016. Applied in PerfLoc competition 2017–1018
System-testing vs. component-testing	System-testing	System-testing	System-testing
Knowledge about the system’s inner-workings	Black-box-testing	Black-box-testing	Black-box-testing
Application-driven T&E approach	Trace-based	Hybrid	Trace-based
Test environment	Building-wide testing	Building-wide testing	Building-wide testing
Building specifications	Large space	Building types classified according to material and size	Classification in one of five building types
Provision of scenarios	-	Scenarios consist of the definition of the experiment process, system, and environment specification	14 scenarios for the localization of persons and objects are proposed by describing motion and building types
Entity to be Localized/Tracked	Person carrying the localization device	Application-dependent	Person, object, robot
Ground truth specification	Realistic measurement resolution	Off-line surveyed points or reference system	Off-line surveyed points or reference system
Specification of path and test points	Points along realistic path	Use-case specific random point sampling	Randomly but uniformly distributed every 50–100 m${}^{2}$ (5–10 m${}^{2}$ for single-family house)
Motion specification	Natural movement of human actor. Standing still at test points	Consideration of motion influence through derived performance metrics	Division of motion into types (walking, crawling, …)
Proposed metrics	Point accuracy	Several performance metrics (point accuracy, energy efficiency, latency, …), derived performance metrics and deployment metrics. Application scenarios are considered to define weights for score calculation	Room and zone accuracy, point accuracy, relative accuracy, latency, setup time, coverage, availability
Metrics applied for absolute position error	75th percentile of horizontal position error	Mean horizontal or spherical position error	Horizontal, vertical and spherical error, covariances, root mean square error (RMSE), mean error, mean of magnitude, variance of magnitude
Consideration of influences	Choice of building and path	RF-interference, environment, mobility, and scalability considered as sensitivity to changes from a reference scenario	Challenging experiments regarding the technology have to be provided. An extensive list of failure modes for various localization technologies is provided

**Table 2:** Exemplary application-dependent scenario for the application of customer tracking in a supermarket. Camera and UWB ILS are considered as localization systems.

	Process Influences			Environment Influences	
**Factors**	**ELT**	**Motion**	**Path**	**Static Objects**	**Lighting Condition**
Specification	Person	Walking	Straight and curves on horizontal plane	Shelves	Daylight and artificial light

**Table 3:** Reference scenario for the *T&E 4iLoc Framework*.

	Process Influences			Environmental Influences	
**Factors**	**ELT**	**Motion**	**Path**	**Static Objects**	**Dynamic Objects**
Specification	Robot	Low velocity, static measurement	Randomly distributed evaluation poses	None	None

**Table 4:** Performance requirements for the process “global navigation”.

Parameter	Absolute Accuracy	Shall/Must	Confidence
Horizontal position	<0.75 m	Must	99.38%
Heading	<60°	Shall	99.38%

**Table 5:** Application-dependent Scenario for an AGV in warehouse operation.

	Process Influences			Environment Influences	
**Factors**	**ELT**	**Motion**	**Path**	**Static Objects**	**Lighting Conditions**
Scenario	Robot	Slow velocity; acceleration/ deceleration	Straight and curves on horizontal plane; driving through aisle	Shelves; vehicles; logistics equipment (altered after mapping)	Daylight; artificial light

**Table 6:** Overview of performance metrics. Metrics referred to in the text are marked bold.

	Mean	Std. Deviation	Median	RMSE	Variance of Magnitude	95th Percentile	99.38th Percentile (4σ)
SICK LOCU UWB							
$\epsilon_{x}$	0.003	0.174	0.006	0.001	0.008	0.268	0.335
$\epsilon_{y}$	0.046	0.259	0.054	0.154	0.022	0.441	0.525
$\epsilon_{\left|x\right|}$	0.149	0.090	0.138	1.637	0.008	0.291	0.366
$\epsilon_{\left|y\right|}$	0.217	0.148	0.224	3.482	0.022	0.469	0.539
$\epsilon_{horizontal}$	0.283	0.138	0.270	5.926	0.019	0.530	0.582
SICK LiDAR-LOC							
$\epsilon_{x}$	**−0.010**	0.062	−0.004	**0.007**	0.002	0.090	0.111
$\epsilon_{y}$	**0.009**	0.064	0.029	**0.006**	0.001	0.090	0.112
$\epsilon_{\left|x\right|}$	**0.049**	0.039	0.042	**0.180**	0.002	0.115	0.154
$\epsilon_{\left|y\right|}$	**0.060**	0.024	0.061	**0.264**	0.001	0.094	0.112
$\epsilon_{horizontal}$	0.085	0.030	0.082	0.532	0.001	**0.131**	**0.166**
$\epsilon_{yaw}$	**−2.209**	**1.819**	−2.155	361.091	2.611	0.615	0.890
$\epsilon_{\left|yaw\right|}$	2.362	1.616	2.155	412.785	2.611	**3.826**	**8.772**
Intel RealSense T265							
$\epsilon_{x}$	0.239	0.288	0.188	4.233	0.061	0.846	0.994
$\epsilon_{y}$	0.633	1.101	0.363	29.662	1.163	3.114	4.879
$\epsilon_{\left|x\right|}$	0.282	0.247	0.193	5.872	0.061	0.846	1.014
$\epsilon_{\left|y\right|}$	0.671	1.078	0.374	33.312	1.163	3.114	4.880
$\epsilon_{horizontal}$	0.817	1.042	0.537	49.391	1.086	3.133	4.881
$\epsilon_{yaw}$	−0.311	2.072	0.011	7.168	1.606	2.320	3.496
$\epsilon_{\left|yaw\right|}$	1.668	1.267	1.475	205.884	1.606	4.203	4.714

*ϵ_x_*: error vector *x*; *ϵ_y_*: error vector *y*; *ϵ*_|*x*|_: absolute error vector *x*; *ϵ*_|*y*|_: absolute error vector *y*; *ϵ_horizontal_*: error vector horizontal; *ϵ_yaw_*: error vector heading; *ϵ*_|*yaw*|_: absolute error vector heading. Length in m; angle in deg.

**Table 7:** Comparison of requirements and respective performance metrics of the SUT for the process “global navigation”.

Parameter	Requirement	SICK LOCU UWB	SICK LiDAR-LOC	Intel RealSense T265 Tracking Camera
Horizontal position error	<0.75 m (must, 4σ-level)	0.58 m	0.17 m	4.88 m
Heading error	<60 °C (shall, 4σ-level)	-	8.77 °C	4.71 °C

## Data Availability

The localization data, the reference data, and the software script used for the empirical validation are provided on GitLab in a publicly available repository [53].

## Abbreviations

AGV	Automated Guided Vehicles
AUC	Application under Consideration
DoF	Degree-of-Freedom
ELT	Entity to be Localized and Tracked
GPS	Global Positioning System
ILS	Indoor Localization System
IMU	Inertial Measurement Unit
IPIN	Conference on Indoor Positioning and Indoor Navigation
IPSN	International Conference on Information Processing in Sensor Networks
ISC	IPIN International Standards Committee
LBS	Location Based Services
LiDAR	Light Detection and Ranging
NIST	US National Institute of Standards and Technology
NLOS	None Line-of-Sight
PTP	Precision Time Protocol
RF	Radio Frequency
RMSE	Root Mean Square Error
ROS	Robot Operating System
RSS	Received Signal Strength
SUT	System Under Test
T&E	Test and Evaluation
UWB	Ultra Wideband
vSLAM	Visual Simultaneous Localization and Mapping
VLP	Visual Light Positioning

## Appendix A. Overview of Publications from IPIN 2021 with Empirical T&E of the Absolute Accuracy

Table A1 provides an overview of the 33 papers from IPIN conference 2021 in which empirical test and evaluation of absolute accuracy was performed. The system type is divided into VLP, RF, magnetic, acoustic, and multiple, depending on the underlying technology of the localization system. The type of ground truth is divided into reference system and other methods. The test environment is divided into hall, building, outdoor, room, small space, and mixed (indoor and outdoor).

**Table A1:** Overview of the 33 publications from IPIN conference 2021, in which empirical test and evaluation of the absolute position accuracy were carried out.

Paper Title	System-Type	Ground Truth	Environment
A Cellular Approach for Large Scale, Machine Learning Based Visible Light Positioning Solutions [19]	VLP	Reference System	Hall
Accurate Multi-Zone UWB TDOA Localization utilizing Cascaded Wireless Clock Synchronization [69]	RF	Reference System	Building
Periodic Extended Kalman Filter to Estimate Rowing Motion Indoors Using a Wearable Ultra-Wideband Ranging Positioning System [70]	RF	Reference System	Hall
Foot-mounted INS for Resilient Real-time Positioning of Soldiers in Non-collaborative Indoor Surroundings [71]	Multiple	Other	Mixed
Quantifying the Degradation of Radio Maps in Wi-Fi Fingerprinting [72]	RF	Other	Building
Measuring Uncertainty in Signal Fingerprinting with Gaussian Processes Going Deep [73]	RF	Reference System	Building
Analysis of IMU and GNSS Data Provided by Xiaomi 8 Smartphone [74]	Multiple	Other	Outdoor
Evolutionary-Inspired Strategy for Particle Distribution Optimization in Auxiliary Particle Filtering Algorithm Based Indoor Positioning [22]	RF	Other	Hall
Comparing and Evaluating Indoor Positioning Techniques [75]	Multiple	Other	Room
Indoor Positioning Using the OpenHPS Framework [76]	Multiple	Other	Building
Anchor Pair Selection for Error Correction in Time Difference of Arrival (TDoA) Ultra Wideband (UWB) Positioning Systems [20]	RF	Reference System	Hall
On the Use of Lookahead to Improve Wi-Fi Fingerprinting Indoor Localization Accuracy [77]	RF	Other	Building
RAD-GAN: Radio Map Anomaly Detection for Fingerprint Indoor Positioning with GAN [78]	RF	Other	Building
v Multipath-Resilient Unsynchronized 2.4 GHz ISM-Band RF Positioning Using Coherent Echos [79]	RF	Reference System	Mixed
Comparative Study of Different BLE Fingerprint Reconstruction Techniques [80]	RF	Other	Building
Finding Optimal BLE Configuration for Indoor Positioning with Consumption Restrictions [81]	RF	Other	Building
MM-Loc: Cross-sensor Indoor Smartphone Location Tracking using Multimodal Deep Neural Networks [82]	Multiple	Other	Building
Received Signal Strength Visible Light Positioning-based Precision Drone Landing System [83]	VLP	Other	Small Space
Experimental Investigation of 5G Positioning Performance Using a mmWave Measurement Setup [21]	RF	Other	Hall
Magnetic Mapping for Robot Navigation in Indoor Environments [84]	Magnetic	Reference System	Hall
Two Efficient and Easy-to-Use NLOS Mitigation Solutions to Indoor 3-D AOA-Based Localization [85]	Acoustic	Other	Hall
POUCET: A Multi-Technology Indoor Positioning Solution for Firefighters and Soldiers [86]	Multiple	Other	Mixed
Adaptive procedure for indoor localization using LoRa devices [87]	RF	Other	Hall
Multidimensional In- and Outdoor Pedestrian Tracking using OpenStreetMap Data [88]	Multiple	Other	Building
Indoor Localization Method For a Microphone Using a Single Speaker [89]	Acoustic	Other	Room
Short-Time and Adaptive Controllable Spot Communication Using COTS Speaker [90]	Acoustic	Other	Small Space
Environment-Aware RSSI Based Positioning Algorithm for Random Angle Interference Cancellation in Visible Light Positioning System [91]	VLP	Reference System	Room
Evaluation of Non-Intrusive Positioning for Public Environments [92]	RF	Reference System	Building
Topology Construction Based on Indoor Crowdsourcing Data using Manifold Learning: Evaluation of Algorithms and Key Parameters [93]	RF	Other	Building
Smartphone Positioning Using an Ambient Light Sensor and Reflected Visible Light [94]	VLP	Other	Small Space
Combined ADS-B and GNSS Indoor Localization [95]	RF	Other	Mixed
Positioning Android Devices in Large Indoor Spaces and Transitioning to Outdoors by Sensor Fusion [96]	Multiple	Other	Building
ProxyFAUG: Proximity-based Fingerprint Augmentation [97]	RF	Reference System	Outdoor

## Appendix B. Overview of the Terminology of the *T&E 4iLoc Framework*

**Table A2:** Terminology of the *T&E 4iLoc Framework*.

Terminology	Explanation
Alignment	Process for converting a SUT coordinate system to the reference coordinate system.
Absolute localization	Localization referring to a large-scale reference. In contrast, relative localization depends on a local frame of reference.
Application-driven influencing factors	Parameters that are expected to influence the performance of a SUT. They can be derived from the AUC and describe influences with a practical character.
Application-dependent T&E	Test and evaluation procedure in which a AUC affects both the test and the evaluation.
Application under Consideration (AUC)	The regarded application or application domain for which a SUT is to be tested and evaluated.
Environment specification	Description of the configuration of a test facility for test and evaluation.
Benchmark	Procedure for executing and evaluating an experiment.
Configuration	A set of parameters that describe the environment or system for an experiment.
Entity to be Localized/Tracked (ELT)	Person, object, vehicle or robot, whose location is to be determined for the AUC or experiment.
Evaluation pose	Pose or position at which the accuracy of the SUT is evaluated.
Evaluation data	Timestamped and aligned pairs of localization data from the SUT and the reference system at the evaluation poses.
Experiment	Physical execution of a *Scenario*.
Experiment data	Continuous localization data from the SUT and the reference system.
Experiment setup	Configuration of the SUT and the environment according to an *Experiment Spec*.
Experiment Spec	Testbed-dependent description of an experiment.
Test facility	Building with a semi-controlled test environment.
Localization data	Timestamped position and heading data.
Location estimate	Timestamped position and heading data provided by the SUT.
Metrics	Indicator of performance for the SUT.
Function module	A set of instructions that serve a higher-level purpose.
Requirements	Set of conditions that a SUT must satisfy to allow reliable realization of an AUC.
Scenario	Set of application-driven influencing factors that abstract the AUC for application-dependent test and evaluation.
Semi-controlled test environments	Indoor facilities that provide a test area with a modifiable environment configuration.
System specification	Description of the hardware and software configuration of a SUT.
System under Test (SUT)	Localization system being tested and evaluated.
Testbed	Combination of test facility, test area, reference system, available ELT and environmental control parameters.
Test and Evaluation (T&E)	Procedure for determining the performance and/or suitability of a localization system.
